# Enhanced Removal of Arsenic from Water by Synthetic Nanocrystalline Iowaite

**DOI:** 10.1038/s41598-017-17903-z

**Published:** 2017-12-13

**Authors:** Qinghai Guo, Yaowu Cao, Zuowei Yin, Zhengyan Yu, Qian Zhao, Zhu Shu

**Affiliations:** 1 State Key Laboratory of Biogeology and Environmental Geology, School of Environmental Studies & Gemological Institute, China University of Geosciences, 430074 Wuhan, Hubei P. R. China; 2 Faculty of Materials Science and Chemistry, China University of Geosciences, 430074 Wuhan, Hubei P. R. China

## Abstract

Nanocrystalline iowaite, a Mg/Fe-based layered double hydroxide (LDH) intercalated with chloride, was synthesized to evaluate its performance for arsenic removal from water and to investigate the contributing dearsenication mechanisms. It is characterized by fast arsenic sorption rates and has a much higher arsenic uptake capacity than other LDHs that are commonly used for water dearsenication. The surface adsorption of the solution arsenic onto the iowaite samples and the anion exchange of the arsenic in solution with chloride, which is originally in the iowaite interlayers, are the primary mechanisms for the uptake of arsenic by iowaite. In addition to the Coulombic attraction between arsenate/arsenite and positively charged layers of iowaite, the inner-sphere complexation of arsenic with Fe (instead of Mg) in the iowaite layers is responsible for the formation of more stable and stronger arsenic bonds, as indicated by both XPS and EXAFS analyses. Specifically, bidentate-binuclear and monodentate-mononuclear As-Fe complexes were detected in the arsenate removal experiments, whereas bidentate-mononuclear, bidentate-binuclear, and monodentate-mononuclear As-Fe complexes were present for the arsenite-treated iowaite samples. This study shows that nanocrystalline iowaite is a promising, low-cost material for arsenic removal from natural arsenic-rich waters or contaminated high-arsenic waters.

## Introduction

Arsenic is a well-known carcinogen in humans and can be found in naturally occurring poor-quality waters and contaminated waters. Among major arsenic species, arsenite is much more toxic than arsenate because the former is characterized by a high affinity for the sulfhydryl groups of amino acids (e.g., cysteine) and is thus capable of inactivating various enzymes involved in intermediate metabolism^1^. The concentrations of arsenic in natural waters are generally below 100 μg/L, but under certain geological conditions, arsenic can accumulate to much higher levels. Indeed, groundwaters containing geogenic arsenic at concentrations exceeding 1000 μg/L have been reported worldwide, especially in India, Bangladesh and northern China^2–4^. In comparison, the anthropogenic arsenic contamination of groundwater is typically induced by the discharge of industrial wastewaters primarily from mining, metal processing, glass and ceramic production, electroplating plants, rubber, fertilizer and semiconductor manufacturing, and fossil-fuel power plants. The arsenic concentrations in mining drainage have been reported to be as high as 850 mg/L^5^. As a result, nearly 50 millions of people worldwide are suffering from the hazards of drinking high-arsenic waters.

The treatment of both arsenic-containing wastewater and high-arsenic drinking water has received substantial attention in countries where arsenic problems occur. Sorption, which is a cost-effective technology, is currently regarded as one of the most promising methods for removing arsenic from aqueous solution. Indeed, sorbents, such as iron-containing compounds, have been widely used for treating high-arsenic waters, and commonly used materials include siderite^6–8^, hematite^9^, goethite^10–12^, magnetite^13^, hydrous ferric oxide^14–16^, and zerovalent iron^17–19^. In recent years, the use of layered double metal hydroxides (LDHs) for purifying arsenic-containing waters has also been reported^20,21^. LDHs, which are also called anion clays, were first discovered as a natural mineral (hydrotalcite) by Hohsteter in 1842 and have a general chemical formula of [M^2+^_1−x_M^3+^_x_(OH)_2_](A^n−^)_x/n_·mH_2_O, where M^2+^ is a divalent cation (typically Mg^2+^, Co^2+^, Ni^2+^, Zn^2+^, or Mn^2+^), M^3+^ is a trivalent cation (typically Al^3+^, Fe^3+^, Cr^3+^, or Ga^3+^), and A^n-^ is an anion (typically OH^−^, Cl^−^, NO_3_^−^, CO_3_^2−^, or SO_4_^2−^)^22^. These hydroxides have large surface areas, huge numbers of exchangeable anions within their interlayer regions and relatively weak interlayer bonding^23^ and, therefore, are effective for the removal of undesirable anions from poor-quality waters, such as fluoride^24,25^, borate^26,27^, nitrate and phosphate^28^, and chromate^29^.

In this study, an iron-bearing LDH, iowaite, was tested for its arsenic uptake capacity and compared with other types of LDHs to determine whether it exhibits a higher affinity with arsenic because of the existence of iron in its layered structure. In fact, the nanocrystalline iowaite synthesized in this study has better performance for arsenic removal than commonly used LDHs and iron-bearing compounds (Table S1). For example, the maximums of arsenic uptake capacity of Mg-Al-NO_3_ LDH and Mg-Al-CO_3_ LDH are 15.8 and 3.75 mg/g, respectively^20^, much lower than that of nanocrystalline iowaite. Another LDH used for arsenic removal from water, hydrocalumite, is characterized by a high capacity of arsenate uptake, but has almost no effects on aqueous arsenite^21^. So, the aim of this study is to examine the extraordinary dearsenication capacity (for both arsenate and arsenite) of nanocrystalline iowaite, provide microscopic evidence for the occurrence of dominant arsenic-iowaite complexes, and reveal the mechanism for arsenic removal by iowaite in solution based on a full understanding of the interactions of iowaite with aqueous arsenic.

## Experimental Section

### Synthesis and Conventional Characterization of Materials

Iowaite (Mg-Fe LDH intercalated with chloride) was prepared via both a previously reported conventional coprecipitation method and a fast coprecipitation and hydrothermal treatment method modified from that proposed by Xu *et al*.^30^. The specific procedures of both methods are described in detail in the Supporting Information (S1) section. The iowaite samples produced by the coprecipitation method (denoted by CP-iowaite) were basically lumpy particles with irregular sizes (from several microns to over two hundred microns) and shapes and rough surfaces. In contrast, those generated via fast coprecipitation followed by hydrothermal treatment (denoted by Nano-iowaite) had a much narrower particle size distribution and a regular crystal structure (see the “Results and Discussion” section for details).

The unreacted iowaite samples and those samples that reacted with arsenic-bearing solutions were characterized using a variety of techniques, including X-ray powder diffraction (XRD), Brunauer-Emmett-Teller specific surface area analysis (BET), photon correlation spectroscopy (PCS), transmission electron microscopy (TEM), scanning electron microscopy equipped with energy dispersive X-ray spectroscopy (SEM/EDX) and X-ray photoelectron spectroscopy (XPS). Detailed descriptions of these techniques are provided in the Supporting Information (S2). The reacted solid samples were also characterized using X-ray absorption near edge structure (XANES) and extended X-ray absorption fine structure (EXAFS) analyses (see the following text for details).

### Batch Tests, Sample Analysis and Sorption Data Evaluation

Unless otherwise stated, all the arsenic removal experiments were performed using the batch sorption technique by reacting 100 mL arsenate or arsenite solutions of various concentrations with 0.2 g of synthetic iowaite. Specifically, the kinetics of arsenic sorption was investigated by reacting iowaite with 0.15, 7.5, 15, 75, 150 and 750 mg/L arsenic solutions, and the isotherm sorption experiments were conducted using initial arsenic concentrations of 0.15, 0.30, 0.75, 1.5, 3.0, 7.5, 15, 30, 75, 150, 300, 450, 600 and 750 mg/L. During the experiments, the sample bottles were sealed and placed in a constant-temperature water bath shaker at 25 °C for a predetermined period. After the sorption experiments, the solution samples were decanted from the bottles, centrifuged and filtered through a 0.22-μm cellulose acetate membrane or a 0.02-μm microporous membrane (for the CP-iowaite-added and Nano-iowaite-added experiments, respectively).

The chemical analysis of chloride in the supernatants was conducted using ion chromatography (IC). The arsenic concentrations were analyzed by atomic fluorescence spectroscopy (AFS) coupled with a flow injection hydride generation system. For selected samples, arsenic was immediately speciated after the experiments using a method reported by Le *et al*.^31^. Briefly, a split of each supernatant sample was passed through a silica-based anion-exchange cartridge. Thus, arsenate was retained by the cartridge, whereas arsenite remained in the filtrate. The anion-exchange cartridge was then eluted by 1 M hydrochloric acid, and the eluent was analyzed for arsenate. Duplicate measurements were performed for all experiments, and the analytical reproducibility for duplicate samples was better than ±5%. The average values of the measured results are reported. The Lagergren first-order model and the pseudo-second-order model were used to fit the data obtained from kinetics studies performed to evaluate the kinetic process of arsenic sorption by iowaite. The data from the isotherm experiments were employed to investigate the sorption isotherms based on the fitting of the Langmuir and Freundlich models (see the Supporting Information (S3) for details).

### XANES and EXAFS Analysis of Sorbed Arsenic

The XANES and EXAFS data collected for sorbed arsenic on the iowaite samples were recorded on beamline BL14W1 at the Shanghai Synchrotron Radiation Facility (SSRF) in China, which employs a Si (111) double crystal monochromator for energy scanning. The ground iowaites were enclosed with Kapton tape in anaerobic chambers with O_2_ levels lower than 1.0 mg/L until the XAS measurement. During the XAS analysis, the samples were sealed with Kapton tape to prevent contact with oxygen and minimize beam-induced oxidation. During the measurement, the electron storage ring operated at 3.5 GeV with a current ranging from 150 to 210 mA. The K-edge spectra of arsenic reacting with iowaite (from −200 to 800 eV relative to the As K-edge of 11867 eV) were recorded in both transmission and fluorescence modes. The absorbance of the incident X-rays was determined using an ionization chamber.

The ATHENA program was used for experimental data processing. All the EXAFS data were normalized by the program using the most intense peak of the first derivative at each elemental foil edge and then transformed from electron energy to a photon-electron wave vector unit (k, Å^−1^). These results were then weighted by k^3^ to generate k^3^χ(k) spectra to better observe the contributions of different shells of the EXAFS oscillation function. Radial structure functions (RSFs) within a k range defined from 3 to 12 Å^−1^ using Hanning windows were further produced by Fourier transformation (FT). The theoretical model fitting was conducted by ARTEMIS to yield the structural parameters, including the interatomic distance (R), coordination number (N) and Debye-Waller factor (σ^2^). The Debye-Waller factors were then employed to adjust the phase and amplitude shift of the EXAFS spectra between the theoretical atomic model and local structure of the iowaite samples used in this study.

## Results and Discussion

### Characteristics of Synthetic Iowaites

The XRD patterns of synthetic CP-iowaite and Nano-iowaite are similar, and both indicate a typical, layered LDH structure (Figure S1a). Sharp and symmetric peaks for the (003) and (006) crystal planes and broader but asymmetric peaks for other planes, which are characteristic of LDHs, were recorded. For Nano-iowaite, the measured d-spacing values of the (003) and (006) planes were 7.93 and 3.95 Å, respectively, and were very similar to those of CP-iowaite (7.97 and 3.96 Å for the (003) and (006) planes, respectively). Elemental analysis revealed that the Mg/Fe molar ratios of both CP-iowaite and Nano-iowaite were almost the same (2.48 and 2.57, respectively) as their designed ratios (i.e., the molar ratio [2.50] of their starting salts). However, Nano-iowaite has a much narrower particle size distribution (166–675 nm with an equivalent hydrodynamic diameter of 386 nm) than CP-iowaite (1.65–666 µm with an equivalent hydrodynamic diameter of 291 µm), as demonstrated by the PCS analysis. TEM analysis revealed that the stable suspensions of Nano-iowaite are monodispersed nanoparticles with a well-shaped hexagonal form (Figure S1b); in contrast, CP-iowaite consists of lumpy particles with irregular sizes and shapes and rough surfaces, as shown by SEM imaging (Figure S1c). Correspondingly, the BET specific surface area of Nano-iowaite is 124.6 m^2^/g, much higher than that of CP-iowaite (5.1 m^2^/g). The chemical formulas, particle sizes, BET surface areas and crystallographic data of both synthetic iowaites are summarized in Table S2.

### Sorption Kinetics and Isotherms

The experimental kinetics results show that Nano-iowaite rapidly removed all the arsenic in the solution (within one hour) for initial arsenic concentrations that did not exceed 15 mg/L. When the initial concentration of arsenic in solution increased beyond this value, a longer equilibrium time was needed for arsenic sorption on Nano-iowaite. In general, a reaction period of 24 h was adequate for all of the initial concentrations tested (from 0.15 to 750 mg/L). The kinetics data of arsenic uptake by Nano-iowaite at initial concentrations of 75, 150 and 750 mg/L were fit to the Lagergren first-order model and the pseudo-second-order model (Figure S2). The correlation coefficients for the pseudo-second-order model were higher than those for the Lagergren first-order model, suggesting that the former better fits the experimental data. Thus, chemical sorption was likely involved in both arsenate and arsenite removal from aqueous solution by Nano-iowaite.

**Figure 1:**
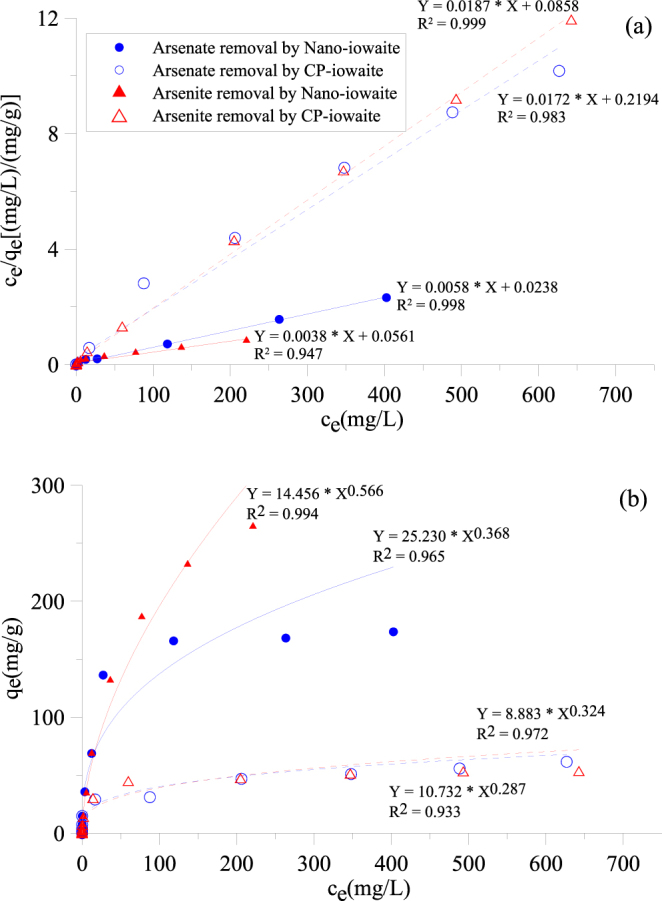
Experimental isotherms of arsenic uptake by the Nano- and CP-iowaite samples and corresponding fits to the Langmuir (**a**) and Freundlich (**b**) models.

The sorption isotherms of arsenic sorbed on Nano-iowaite and CP-iowaite were well fitted by the Freundlich and Langmuir models (Fig. 1), indicating that Nano-iowaite has much higher uptake capacities for both arsenate and arsenite than CP-iowaite. By extrapolating the Langmuir equation, the sorption maxima of Nano-iowaite for arsenate and arsenite were calculated to be 172.4 and 263.2 mg/g, respectively, whereas those of CP-iowaite were 58.1 and 53.5 mg/g, respectively. This difference can be attributed to the much narrower particle size distribution and larger surface area of Nano-iowaite.

### Reaction of Nano-iowaite and Arsenic in Solution

The XRD analysis results for the reacted Nano-iowaite samples include iowaite peaks at initial arsenic concentrations lower than 750 mg/L (Figure S3). At an arsenate concentration of 750 mg/L, however, a small peak corresponding to a compound other than iowaite was identified (Figure S4). Further inspection indicates that angelellite (Fe_4_(AsO_4_)_2_O_3_) or hornesite (Mg_3_(AsO_4_)_2_·8H_2_O) formed via the coprecipitation of As, which was originally in solution, with Fe or Mg from iowaite dissolution. Nevertheless, the amount of this newly formed mineral is miniscule, as indicated by its weak XRD peak.

Thus, the contribution of the formation of new arsenic-bearing minerals to the removal of arsenic from the solution is very limited. According to the analyses of arsenic and chloride in solution after the sorption reaction, the increment of the solution chloride concentration is positively related to the decrement of the solution arsenate or arsenite concentration (Figure S5). Thus, the anion exchange between the arsenic in solution and the chloride that was originally intercalated into the interlayers of the iowaite samples was the primary mechanism for the uptake of arsenic by iowaite. The sorbed arsenic basically binds to the iowaite surface or within its interlayers by forming an outer-sphere complex; that is, the driving force for arsenic sorption onto/into iowaite is likely the Coulombic attraction between arsenate or arsenite and the positively charged layers of iowaite. However, it cannot be ruled out that the inner-sphere complexation of arsenate or arsenite with certain functional groups on the layers of iowaite may also occur and form a more stable and stronger arsenic bond. The latter mechanism will be discussed in detail later.

### Arsenite Oxidation during its Removal by Iowaite

In this study, all the dearsenication experiments were conducted under oxic conditions, and a part of the arsenite in solution was typically oxidized to arsenate (between 10% and 30%; see Table S3) during the reaction with the iowaite samples. However, no arsenite was detected in the solutions recovered from the arsenate-iowaite reaction systems.

**Figure 2:**
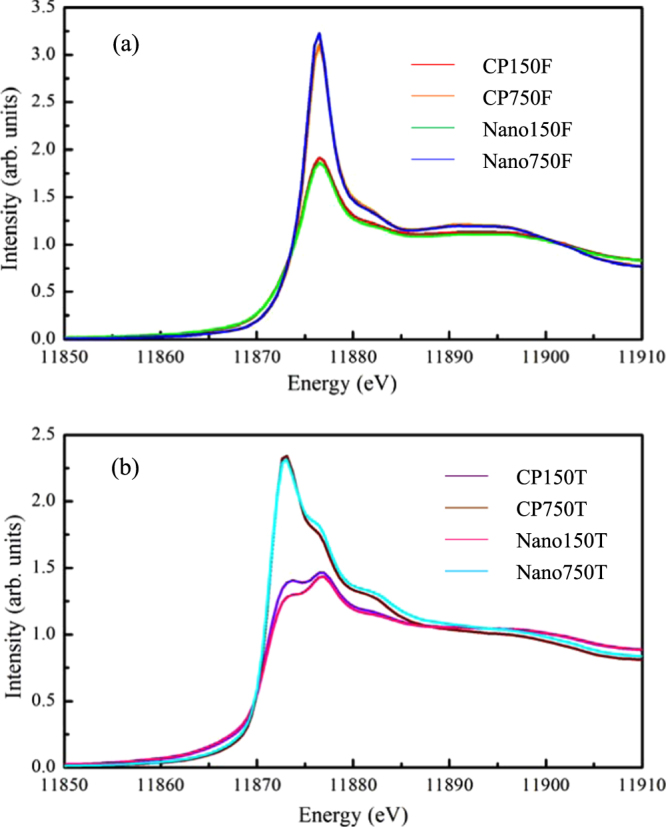
K-edge XANES spectra of arsenic for the iowaite samples reacted with arsenate- (**a**) and arsenite- (**b**) bearing solutions. The sample labels used in this figure are the same as those used in Tables 1 and 2.

**Figure 3:**
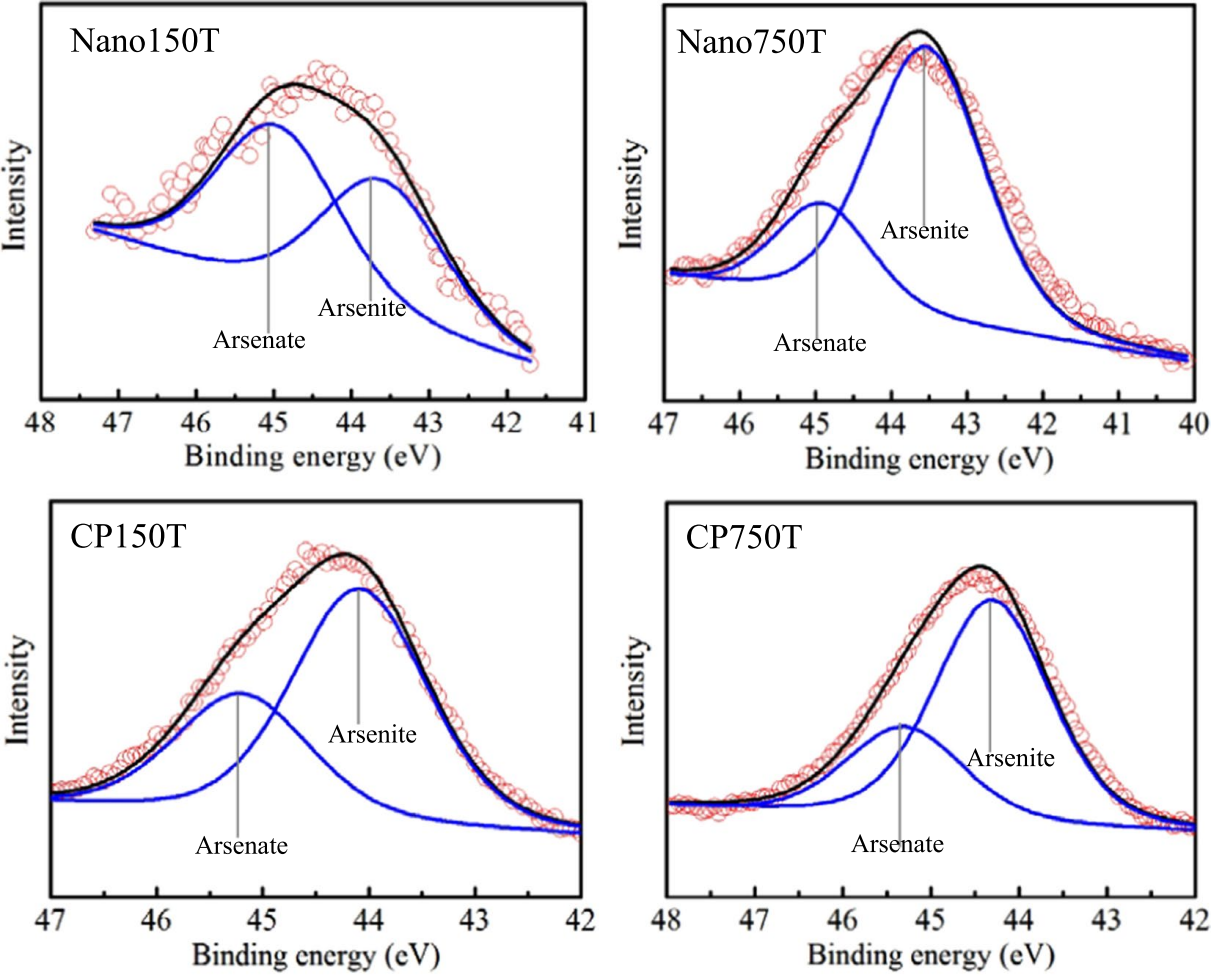
XPS spectra of As 3d for the reacted Nano- and CP-iowaite samples. The red dotted lines represent the original experimental results, and the solid lines denote their fits. The sample labels used in this figure are the same as those used in Tables 1 and 2.

Figure 2a and b show the XANES data for arsenate- and arsenite-treated samples, respectively. Similarly, no redox transformation of the sorbed arsenate was observed for the arsenate-treated samples, as indicated by the absorption maxima occurring at 11876.5 eV (an energy position typically indicative of arsenate). However, as expected, the sorbed arsenite was partially oxidized to arsenate on both Nano-iowaite and CP-iowaite, leading to an evident shift in the X-ray absorption edge from 11873 eV to 11876.5 eV. The partial oxidation of arsenite to arsenate on the sorbents was also confirmed by their XPS spectra (Fig. 3). These spectra further indicate that much more arsenite was oxidized to arsenate when the initial solution arsenic concentration was 150 mg/L (47.7% and 34.2% of the sorbed arsenic oxidized on Nano- and CP-iowaite, respectively). In comparison, the concentration percentages of oxidized arsenic were 23.3% and 28.5% for Nano- and CP-iowaite, respectively, when the initial solution arsenic concentration was 750 mg/L (Table S3).

This arsenite oxidation was not likely induced by the X-ray beam during XAS analysis, in view that the arsenite in solution was also oxidized as indicated by the measurements of solution arsenic speciation after the sorption experiments. The ratios of oxidized arsenic to total arsenic in the solutions are similar to those on the sorbents (Table S3). In this study, the mechanism for the oxidation of arsenite is substantially different from that reported for the As(III)-Fe(II)-O_2_ and As(III)-Fe(0)-O_2_ systems, in which Fenton-like processes occurred, produced strongly oxidizing radical species, and rapidly oxidized arsenite both in solution and on sorbent^6,32–35^. Fe(II) and zero-valent iron were not used or generated in the reaction systems in this study. Hence, the most likely reason for the oxidation of arsenite during its sorption by iowaites is that arsenite auto-oxidizes at a high solution pH of 10–11. The high pH values of the solutions were attributable to the dissolution of iowaite, which released OH^-^; therefore, the solution arsenite was exposed to a high-pH environment. This rapid oxidation of arsenite at pH values exceeding the first pKa for arsenite (pH = 9.2) has also been commonly reported in previous studies^36–39^. Moreover, other factors may have been responsible for the formation of mixed arsenite/arsenate speciation in arsenite-iowaite reaction systems. Manning *et al*. (2002) reported that maghemite and hematite are capable of oxidizing the coexisting arsenite phases. However, although Fe-bearing minerals (e.g., angelellite) may have formed in this experimental study, iron oxides and oxyhydroxides were not detected after the reaction between the iowaite and arsenic solutions (even at a high initial concentration of 750 mg/L). Thus, the formation of new minerals did not contribute to the occurrence of arsenite oxidation.

### Microscopic Analysis of the Mechanism for Arsenic Removal by As-Fe Complexation

As mentioned earlier, Nano-iowaite has a high arsenic uptake capacity, as confirmed by the XPS results. The reacted Nano-iowaite samples exhibited intense As 3d peaks, whereas the unreacted sample did not (Figure S6a). In comparison, the intensity of Cl 2p peaks of the reacted Nano-iowaite samples are much lower than those of the unreacted sample (Figure S6b), suggesting that significant interlayer Cl was substituted by solution As. The removal of As from the solution by Nano-iowaite, however, seems unrelated to the Mg atoms in its layered structure because of the minimal difference in the Mg 2p spectra of the solid samples before and after arsenic sorption (Figure S6c). Instead, the binding energy values of Fe in the unreacted Nano-iowaite sample (726.45 eV for the Fe 2p 1/2 peak and 712.35 eV for the Fe 2p 3/2peak) shift to lower values after arsenic sorption (Figure S7), clearly indicating the significant role of Fe atoms in As uptake. The changes in the Mg and Fe spectra of the CP-iowaite samples upon As sorption (figures not presented) were similar to those of the Nano-iowaite samples, implying the complexation of As with Fe rather than Mg. Thus, the presence of Fe instead of Mg in the structure of iowaite is critical for high As uptake capacity.

**Figure 4:**
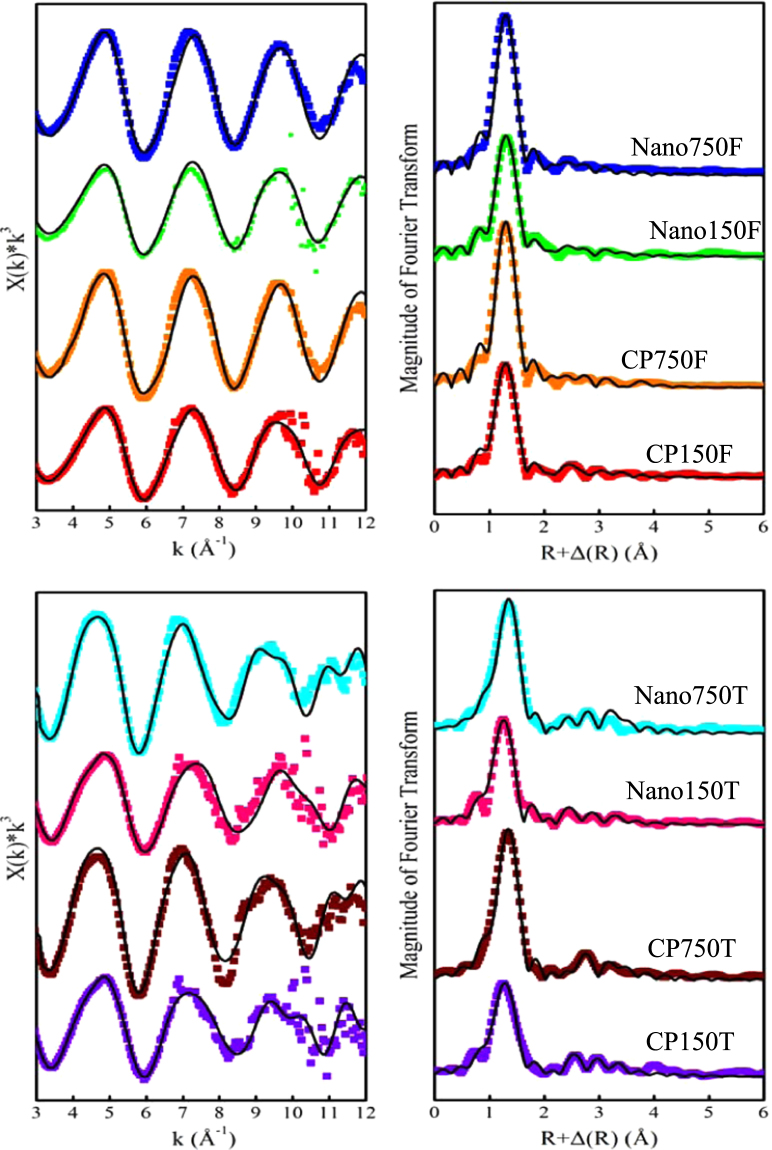
*k* ^*3*^ *Χ(k)* spectra (left) and corresponding FT results (right) of the EXAFS signals for arsenic sorbed onto iowaite. The dotted and solid lines represent experimental results and fitted data derived from the theoretical EXAFS function with ARTEMIS, respectively. The peak positions in the FT spectra correspond to the distances of the atomic shells without a phase shift correction. The sample labels used in this figure are the same as those used in Tables 1 and 2.

Figure 4 shows the experimental radial distribution functions (RDFs) that were produced from the FT EXAFS data of the reacted iowaite samples and the theoretical EXAFS expressions fit to these experimental data. The experimental results and fitted data are displayed as dotted and solid lines, respectively. The fitted parameters are presented in Tables 1 and 2 for arsenate- and arsenite-treatment experiments, respectively.

**Table 1:** Structural Parameters of Arsenate-treated Iowaite Determined by the EXAFS Analysis for the Local Coordination Environment of Arsenic Sorbed onto the Iowaite Samples.

Sample ID	Interatomic shell	Interatomic distance (R/Å)	Coordination number (CN)	Debye-Waller factor (σ^2^/Å^2^)
Nano150F	As-O	1.69	4.0	0.0029
	As-O-O	3.11	12.0	0.0026
	As-Fe_2_	3.33	2.0	0.0211
	As-Fe_3_	3.51	1.0	0.0096
CP150F	As-O	1.69	4.0	0.0036
	As-O-O	3.07	12.0	0.0006
	As-Fe_2_	3.31	2.0	0.0189
	As-Fe_3_	3.44	1.0	0.0073
Nano750F	As-O	1.69	4.0	0.0021
	As-O-O	3.08	12.0	0.0002
	As-Fe_2_	3.34	2.0	0.0233
	As-Fe_3_	3.47	1.0	0.0130
CP750F	As-O	1.69	4.0	0.0018
	As-O-O	3.11	12.0	0.0002
	As-Fe_2_	3.35	2.0	0.0518
	As-Fe_3_	3.50	1.0	0.0165

The sample labels used in the table are as follows: “CP” indicates iowaite synthesized by a conventional coprecipitation method; “Nano” refers to nanocrystalline iowaite generated via fast coprecipitation followed by hydrothermal treatment; the capital letter “F” refers to arsenate-treated samples, and the numbers before “F” (150 and 750) represent the initial arsenic concentrations of the solutions (150 and 750 mg/L, respectively).

**Table 2:** Structural Parameters of Arsenite-treated Iowaite from the EXAFS Analysis for the Local Coordination Environment of Arsenic Sorbed onto the Iowaite Samples.

Sample ID	Interatomic shell	Interatomic distance (R/Å)	Coordination number (CN)	Debye-Waller factor (σ^2^/Å^2^)
Nano150T	As-O	1.67	3.7	0.0057
	As-O-O	3.18	10.0	0.0191
	As-Fe_1_	3.08	1.0	0.0105
	As-Fe_2_	3.40	2.0	0.0094
	As-Fe_3_	3.56	1.2	0.0052
CP150T	As-O	1.70	3.7	0.0072
	As-O-O	3.17	10.0	0.0056
	As-Fe_1_	3.05	1.0	0.0194
	As-Fe_2_	3.39	2.0	0.0052
	As-Fe_3_	3.52	1.0	0.0003
Nano750T	As-O	1.75	3.5	0.0057
	As-O-O	3.19	8.0	0.0100
	As-Fe_1_	3.05	1.0	0.0091
	As-Fe_2_	3.32	2.0	0.0078
	As-Fe_3_	3.53	1.0	0.0023
CP750T	As-O	1.74	3.5	0.0044
	As-O-O	3.19	8.0	0.0310
	As-Fe_1_	3.05	1.0	0.0192
	As-Fe_2_	3.33	2.0	0.0057
	As-Fe_3_	3.47	1.0	0.0006

The sample labels used in the table are as follows: “CP” indicates iowaite synthesized by conventional coprecipitation method; “Nano” refers to nanocrystalline iowaite generated via fast coprecipitation followed by hydrothermal treatment; the capital letter “T” refers to arsenite-treated samples, and the numbers before “T” (150 and 750) represent the initial arsenic concentrations of the solutions (150 and 750 mg/L, respectively).

The spectra of all arsenate-treated samples are dominated by the contribution from the As-O first shell of 4.0 oxygen atoms at an As-O interatomic distance of 1.69 Å, which matched well with the expected As(V)-O distance in the AsO_4_ tetrahedron. The As-Fe pairs at two different distances were used to successfully fit the second-neighbor contributions to the EXAFS data. Specifically, these As-Fe shells are composed of 2.0 Fe atoms (As-Fe2) at approximately 3.31–3.35 Å and 1.0 Fe atoms (As-Fe3) at approximately 3.44–3.51 Å. The As-Fe distances of 3.31–3.35 Å can be attributed to a bidentate-binuclear arsenate complex and are very similar to those of the As-Fe2 shell, as previously reported for other iron-bearing materials used for the treatment of solution arsenate, such as siderite^6,40^, goethite^37^, and green rust^41^. Similarly, the arsenic-Fe distances of 3.44–3.51 Å obtained in this study are ascribed to a monodentate-mononuclear As-Fe complex, in which the AsO_4_ tetrahedron is attached to the adjacent apex of the edge-sharing Fe octahedral. Moreover, a multiple-scattering (MS) contribution, which corresponds to twelve As-O-O-As paths within the AsO_4_ tetrahedron, was also considered because it helped improve the fit to the EXAFS data (Fig. 4 and Table 1).

For the arsenite-treated samples, the first coordination shell surrounding arsenic was fit by 3.5–3.7 oxygen atoms at As-O distances of 1.67–1.75 Å (different from the expected As(III)-O distance in the AsO_3_ pyramidal molecule of 1.79 Å), which implies that a mixed arsenite/arsenate speciation in the reacted iowaite samples formed via partial arsenite oxidation. Further inspection shows that the iowaite samples that reacted with the 750 mg/L arsenic solution have fewer arsenic-surrounded oxygen atoms and longer As-O interatomic distances than those that reacted with the 150 mg/L arsenic solution, indicating that less arsenite is oxidized in the former case. In addition to the As-O shell, three As-Fe shells and a contribution from six As-O-O-As paths within the AsO_3_ pyramid gave a reasonable fit to the EXAFS data (Fig. 4 and Table 2). The three As-Fe shells are 1.0 Fe atoms at 3.05–3.08 Å, 2.0 Fe atoms at 3.32–3.40 Å, and 1.0–1.2 Fe atoms at 3.47–3.56 Å and correspond to bidentate-mononuclear, bidentate-binuclear, and monodentate-mononuclear As-Fe complexes, respectively.

The XAS analysis indicated that both binuclear and mononuclear complexes are present for the reacted iowaite samples. The binuclear corner-sharing (2 C) AsO_4_ complexes have been reported to be much less energetic than both the mononuclear edge-sharing (2E) and corner-sharing (1 V) AsO_4_ complexes for Fe(III) oxides or hydroxides, such as goethite, and the formation of the former is therefore more favorable^42,43^. Thus, the binuclear As-Fe complex is energetically favored and can be formed regardless of the coverage of arsenic on the sorbent surface. In contrast, the mononuclear As-Fe complex may occur only when the surface coverage of arsenic is high. In this study, the iowaite samples utilized for XAS analyses were those that were reacted with 150 and 750 mg/L arsenic solutions and had high arsenic loadings (Table S4). Consequently, both binuclear and mononuclear complexes were detected in these reacted iowaite samples. This differs from a previous study conducted by Farquhar *et al*.^44^, in which arsenite-sorbed goethite was characterized by a much lower arsenic coverage and dominated by binuclear As-Fe complexes.

### Broader Discussion of Future Applications

The experimental work conducted in this study indicates that iowaite, an iron-bearing LDH, can be used to effectively remove arsenic from aqueous solution. The nanocrystallization of iowaite via fast coprecipitation followed by hydrothermal treatment helps to further enhance its dearsenication capacity. Nano-iowaite also performed better than many other commonly used sorbents with respect to solution dearsenication under similar experimental conditions (including the mass ratio of solution arsenic to sorbent), as shown in Table S1.

The primary mechanism for arsenic removal by Nano-iowaite is anion exchange between the arsenic in the solution and the original chloride in the iowaite structure. The intercalated arsenic was bound via the Coulombic attraction of the positively charged layers. Additionally, the stronger inner-sphere complexation of arsenate or arsenite with Fe in the iowaite layers was responsible for its superior dearsenication capacity relative to other LDHs, such as hydrotalcite and hydrocalumite. During the formation of As-Fe complexes, the original Fe-OH ligand was broken, and various Fe-O-As ligands were generated. The experimental results indicate that Nano-iowaite is a promising material for the treatment of both industrial wastewaters with arsenic concentrations over several hundreds of mg/L and contaminated natural waters with a comparatively lower range of arsenic concentrations (typically from several hundreds of µg/L to several mg/L). For industrial-scale applications, Nano-iowaite must be synthesized as microspheres with a macro porous structure or as a homogeneous mixture with other chemically stable materials, such as quartz sand, so that the material has sufficient diffusion pathways for arsenic-bearing waters. In terms of water dearsenication, one limit of Nano-iowaite is that the final solution is usually alkaline (pH 10–11). However, this may be advantageous when Nano-iowaite is used to treat acidic arsenic-rich waters from, for example, acid geothermal discharge or acid mine drainage. By carefully designing the treatment conditions, the final solution pH could be within the neutral range, and arsenic could be efficiently removed from water. One problem possibly occurring during the treatment of acid waters by Nano-iowaite is that it may be not as stable as in alkaline solutions. So a preliminary stability test on Nano-iowaite at pHs from 2 to 6 was carried out. The results show that there are only iowaite peaks in the XRD patterns of all the reacted solid samples (Figure S8). Nevertheless, the mass percentage of remained Nano-iowaite decreases from 99.6% at pH = 6 to 97.0% at pH = 2 (see the details in Table S5), implying that at acidic pHs, iowaite is indeed slightly more unstable than at alkaline pHs. A more systematic test on the stability of Nano-iowaite at wider ranges of pH and arsenic concentration needs to be performed before it will be put into practice in acid water treatment.

## Electronic supplementary material

Supplementary Information
