# Supplementary material for: Enhanced Removal of Arsenic from Water by Synthetic Nanocrystalline Iowaite
Source: Sci Rep. 2017 Dec 13;7:17546. doi: 10.1038/s41598-017-17903-z (PMC5727487; doi:10.1038/s41598-017-17903-z)
Supplement: Supplementary file 1 — Supplementary Information [file 41598_2017_17903_MOESM1_ESM.pdf]

Supplementary Information

Enhanced Removal of Arsenic from Water by Synthetic Nanocrystalline Iowaite

Qinghai Guo, Yaowu Cao, Zuowei Yin, Zhengyan Yu, Qian Zhao, Zhu Shu

S1. Synthesis of iowaites

Non-nanocrystalline Mg-Fe(III) LDH was synthesized by a conventional coprecipitation method (denoted by CP-iowaite). An aqueous solution containing 1.0-mol/L magnesium chloride ($MgCl_2 \cdot 6H_2O$) and 0.5-mol/L ferric chloride ($FeCl_3 \cdot 6H_2O$) (the ratio of Mg to Fe(III) in the solution was 2:1) was added dropwise to an 8.0-mol/L NaOH solution under vigorous mechanical stirring. During this process, the temperature was maintained at 75 ± 3 °C. After the addition of the $MgCl_2$ and $FeCl_3$ solution, a thick slurry was obtained and heated for 2 h at 95 ± 3 °C for aging. The solid products were separated by centrifugation (4000 r/min for 10 min) and washed several times with Nanopure water to a pH of 7 – 8. Finally, the solids were dried in a vacuum desiccator at room temperature for further use.

The nanocrystalline Mg-Fe(III) LDH (denoted by Nano-iowaite) was synthesized via fast coprecipitation followed by hydrothermal treatment. A mixed salt solution comprising 20 mmol of $MgCl_2 \cdot 6H_2O$ and 10 mmol of $FeCl_3 \cdot 6H_2O$ was promptly added to 50 mL of 1.4 M NaOH with nitrogen purging and vigorous stirring. The iowaite slurry was centrifuged at 4000 r/min for 10 min, washed three times with Nanopure water, and finally dispersed in Nanopure water. The resulting aqueous suspension was then hydrothermally treated in a stainless steel autoclave at 80 °C for 4 h. After air-cooling, a stable homogeneous iowaite suspension (the product) was further freeze-dried at -20 °C to avoid aggregation. The synthesized iowaite samples were kept in a desiccator to prevent the possible sorption of atmospheric CO_2 and moisture.

S2. Solid sample characterization

XRD was applied to the as-prepared and reacted iowaite samples using an X’Pert PRO DY2198 diffractometer with Cu Kα radiation (λ = 1.541 Å). The XRD patterns were collected in θ-2θ mode between $5° \leq 2\theta \leq 65°$ with a 2θ step of 0.033°.

The BET specific surface areas of the reacted iowaite samples were measured by the N_2-adsorption/desorption method at liquid nitrogen temperature with a Micromeritics ASAP2020 instrument.

The TEM analysis of synthetic Nano-iowaite was performed on a JEOL JSM-2010 TEM with an acceleration voltage of 200 kV. Prior to TEM imaging, the iowaite nanoparticles were dispersed in alcohol under ultrasonication for 10 min, and a droplet was subsequently dropped on a carbon-coated copper grid. The field-limiting apertures for selected-area electron diffraction were 5 - 60 μm in diameter, and the TEM image was collected via a 20-μm, high-contrast objective aperture.

Portions of the reacted and unreacted iowaite samples were analyzed with an FEI Quanta200 Scanning Electron Microscope (SEM) equipped with EDX. Images were taken at 20-kV excitation to highlight the compositional variations in the samples. EDX was performed at 20 kV to determine the elemental composition.

XPS was conducted on a Kratos AXIS Ultra spectrometer with a monochromatic Al Kα X-ray source operated at 15 kV (150 W and 10 mA) and in fixed-analyzer transmission mode. The energy scale of the XPS spectra was calibrated using the binding energy of the C 1s peak (284.6 eV) as the reference line. The analysis was conducted under ultra-high vacuum (10^{-10} mbar), and AVANTAGE software was used to interpret the obtained data.

S3. Sorption data processing

From the arsenic concentrations of the equilibrated solutions (c_e in mg/L), the sorbed arsenic concentrations (q_e in mg/g) and the percent removal of arsenic (R in %) were calculated using equations (1) and (2).

$$q_e = (c_0 - c_e) * v / m \quad (1)$$

$$R = \frac{c_0 - c_e}{c_0} * 100\ \% \quad (2)$$

where c_0 and c_e (in mg/L) are the initial and equilibrium concentrations of arsenic in solution, respectively; v (in L) denotes the volume of the arsenic solution; and m (in g) is the mass of the sorbent.

The kinetics of arsenic sorption by iowaites was investigated by performing reactions with 0.15, 7.5, 15, 75, 150 and 750 mg/L arsenic solutions. Two different

kinetic models were used to fit the experimental data: (1) the Lagergren first-order model and (2) the pseudo-second-order model. The Lagergren first-order model [1] was developed for irreversible sorption in solid/liquid systems and is described by the following rate expression:

$$q_t = q_e(1 - e^{-k_1 t}) \tag{3}$$

where q_e (mg/g) is the amount of sorbate concentration in the solid at equilibrium, q_t (mg/g) is the amount of sorbate in the solid at time t, and k_1 is the Lagergren first-order rate constant. By plotting $\ln(q_e - q_t)$ against t, a linear relationship should be obtained:

$$\ln(q_e - q_t) = \ln(q_e) - k_1 t \tag{4}$$

The pseudo-second-order model [2] is based on the assumption that chemical sorption is one of the factors controlling the sorption kinetics. The pertinent rate expression is:

$$\frac{t}{q_t} = \frac{1}{k_2 q_e^2} + \frac{t}{q_e} \tag{5}$$

where k_2 is the pseudo-second-order rate constant.

In the isotherm sorption experiments performed to investigate the effect of arsenic concentration on its uptake by iowaite samples, the initial arsenic concentrations were 0.15, 0.30, 0.75, 1.5, 3.0, 7.5, 15, 30, 75, 150, 300, 450, 600, and 750 mg/L. The isotherm sorption data (q_e vs. c_e) were fit to both the Langmuir and Freundlich isotherm models (equations (6) and (7), respectively).

$$q_e = \frac{q_0 b c_e}{1 + b c_e} \tag{6}$$

$$q_e = K c_e^n \tag{7}$$

where q_0 is the maximum sorption capacity (mg/g); b is a constant related to the sorption energy (L/mg); K is the Freundlich constant, which indicates the sorption capacity of the sorbent [(mg/g) (L/mg)^n]; and n reflects the sorption intensity.

Table S1. Arsenic Uptake Capacities of a Variety of Materials.

Sorbent	Sorption capacity (mg/g)		Initial As concentration (mg/L)	Ratio of sorbent to solution (g/L)	Reference
	arsenite	arsenate			
Calcined iowaite	3.4	3.7	7.5	2	[3]
Mg-Al-NO_3 LDH	-	15.8	50	1	[4]
Mg-Al-CO_3 LDH	-	3.75	50	1	[4]
Iron-Oxide Coated Sand	-	0.20	3.5	20	[5]
Ferrihydrite	40	-	250	25	[6]
Hematite	1	-	250	25	[6]
Goethite	5	-	250	25	[6]
Lepidocrocite	7	-	250	25	[6]
Granular iron hydroxide	-	5.2	100	4	[7]
Hematite	0.2	-	10	40	[8]
Nanocrystalline iowaite	0.75	0.75	1.5	2	This study
Nanocrystalline iowaite	3.7	3.8	7.5	2	This study
Nanocrystalline iowaite	68.8	68.9	150	2	This study
Nanocrystalline iowaite	264.5	173.7	750	2	This study

Table S2. Chemical Formulas, Particle Sizes (nm for Nano-iowaite and μm for CP-iowaite), BET Surface Areas (m^2/g), Lattice Parameters (Å) and Crystal Sizes (nm) of the Synthetic Iowaite Samples.

Synthetic sorbent	Chemical formula	Textural property		Lattice parameter		Crystal size	
		Particle sizes	BET surface area	a	c	a-axis	c-axis
CP-iowaite	$Mg_{2.48}Fe(OH)_{6.96}Cl_{0.63}(CO_3)_{0.18}$	1.65 - 666 μm	5.1	3.112	23.899	48.343	12.971
Nano-iowaite	$Mg_{2.57}Fe(OH)_{7.14}Cl_{0.98}(CO_3)_{0.01}$	166 - 675 nm	124.6	3.115	23.792	33.229	7.783

Lattice parameter "a" (equivalent to 2 × d(110)) represents the average metal-metal distance inside the brucite-like sheets of synthetic iowaites, and "c", expressed as 3 × d(003), indicates the distance between layers affected by the sizes and charges of the interlayer anions. The crystal sizes of the iowaite samples were calculated using the Scherrer equation.

Table S3. Arsenic Speciation Analyses of the Solutions and the Sorbents after the Sorption Reaction.

**No.**	**Concentration in solution**				**Concentration percent in solid phase**	
	**Arsenite (mg/L)**	**Arsenate (mg/L)**	**Arsenite (%)**	**Arsenate (%)**	**Arsenite (%)**	**Arsenate (%)**
CP-Ta	0.000	0.000	N/A	N/A		
CP-Tb	0.000	0.000	N/A	N/A		
CP-Tc	0.000	0.000	N/A	N/A		
CP-Td	0.001	0.000	100	0		
CP-Te	0.014	0.000	100	0		
CP-Tf	0.072	0.019	79.6	20.4		
CP-Tg	0.254	0.039	86.6	13.4		
CP-Th	1.430	0.170	89.4	10.6		
CP-Ti	12.24	1.893	86.6	13.4		
CP-Tj	28.36	31.37	47.5	52.5	65.8	34.2
CP-Tk	155.8	49.14	76.0	24.0		
CP-Tl	221.9	124.9	64.0	36.0		
CP-Tm	362.7	130.2	73.6	26.4		
CP-Tn	493.8	148.7	76.9	23.1	71.5	28.5
**No.**	**Concentration in solution**				**Concentration percent in solid phase**	
	**Arsenite (mg/L)**	**Arsenate (mg/L)**	**Arsenite (%)**	**Arsenate (%)**	**Arsenite (%)**	**Arsenate (%)**
Nano-Ta	0.000	0.000	N/A	N/A		
Nano-Tb	0.000	0.000	N/A	N/A		
Nano-Tc	0.000	0.000	N/A	N/A		
Nano-Td	0.000	0.000	N/A	N/A		
Nano-Te	0.014	0.002	85.4	14.6		
Nano-Tf	0.090	0.008	91.9	8.1		
Nano-Tg	0.199	0.047	80.9	19.1		
Nano-Th	1.331	0.248	84.3	15.7		
Nano-Ti	3.695	1.911	65.9	34.1		
Nano-Tj	7.898	4.542	63.5	36.5	52.3	47.7
Nano-Tk	28.72	7.623	79.0	21.0		
Nano-Tl	54.31	22.89	70.4	29.6		
Nano-Tm	111.9	24.61	82.0	18.0		
Nano-Tn	170.4	50.58	77.1	22.9	76.7	23.3

The percentages of arsenite and arsenate in the reacted iowaite samples were estimated based on the XPS analyses. The sample labels used in the table are as follows: “CP” indicates iowaite synthesized by a conventional coprecipitation method; “Nano” refers to nanocrystalline iowaite generated via fast coprecipitation followed by hydrothermal treatment; the capital letter “T” refers to arsenite-treated samples, and the lowercase letters following “T” (a, b, c, d, e, f, g, h, i, j, k, l, m and n) represent the initial arsenic concentrations of the solutions (a: 150 μg/L; b: 300 μg/L; c: 750 μg/L; d: 1500 μg/L; e: 3000 μg/L; f: 7500 μg/L; g: 15 mg/L; h: 30 mg/L; i: 75 mg/L; j: 150 mg/L; k: 300 mg/L; l: 450 mg/L; m: 600 mg/L; n: 750 mg/L).

Table S4. Experimental Isotherm Data of Arsenite and Arsenate Removal by CP-iowaite and Nano-iowaite. c_0: initial solution arsenic concentration; c_e: solution arsenic concentration at equilibrium; q_e: sorbed arsenic concentration; R: percent removal of arsenic.

**No.**	**c_0 (mg/L)**	**c_e (mg/L)**	**R (%)**	**q_e (mg/g)**	**No.**	**c_0 (mg/L)**	**c_e (mg/L)**	**R (%)**	**q_e (mg/g)**
CP-Ta	0.15	0	100	0.075	CP-Fa	0.15	0	100	0.075
CP-Tb	0.30	0	100	0.150	CP-Fb	0.30	0	100	0.150
CP-Tc	0.75	0	100	0.375	CP-Fc	0.75	0	100	0.375
CP-Td	1.5	0.001	99.97	0.750	CP-Fd	1.5	0	100	0.750
CP-Te	3.0	0.014	99.5	1.493	CP-Fe	3.0	0.008	99.7	1.496
CP-Tf	7.5	0.091	98.8	3.705	CP-Ff	7.5	0.020	99.7	3.740
CP-Tg	15	0.294	98.0	7.353	CP-Fg	15	0.063	99.6	7.468
CP-Th	30	1.600	94.7	14.20	CP-Fh	30	0.137	99.5	14.93
CP-Ti	75	14.13	81.2	30.43	CP-Fi	75	16.77	77.6	29.12
CP-Tj	150	59.73	60.2	45.14	CP-Fj	150	87.67	41.6	31.16
CP-Tk	300	204.9	31.7	47.53	CP-Fk	300	206.0	31.3	46.98
CP-Tl	450	346.9	22.9	51.56	CP-Fl	450	347.9	22.7	51.05
CP-Tm	600	493.0	17.8	53.53	CP-Fm	600	488.3	18.6	55.88
CP-Tn	750	642.5	14.3	53.75	CP-Fn	750	626.8	16.4	61.61
**No.**	**c_0 (mg/L)**	**c_e (mg/L)**	**R (%)**	**q_e (mg/g)**	**No.**	**c_0 (mg/L)**	**c_e (mg/L)**	**R (%)**	**q_e (mg/g)**
Nano-Ta	0.15	0	100	0.075	Nano-Fa	0.15	0	100	0.075
Nano-Tb	0.30	0	100	0.150	Nano-Fb	0.30	0	100	0.150
Nano-Tc	0.75	0	100	0.375	Nano-Fc	0.75	0	100	0.375
Nano-Td	1.5	0	100	0.750	Nano-Fd	1.5	0	100	0.750
Nano-Te	3.0	0.016	99.5	1.492	Nano-Fe	3.0	0	100	1.500
Nano-Tf	7.5	0.098	98.7	3.701	Nano-Ff	7.5	0	100	3.750
Nano-Tg	15	0.246	98.4	7.377	Nano-Fg	15	0.045	99.7	7.478
Nano-Th	30	1.580	94.7	14.21	Nano-Fh	30	0.283	99.1	14.86
Nano-Ti	75	5.606	92.5	34.70	Nano-Fi	75	3.469	95.4	35.77
Nano-Tj	150	12.44	91.7	68.78	Nano-Fj	150	12.31	91.8	68.85
Nano-Tk	300	36.34	87.9	131.8	Nano-Fk	300	27.21	90.9	136.4
Nano-Tl	450	77.20	82.8	186.4	Nano-Fl	450	118.3	73.7	165.8
Nano-Tm	600	136.5	77.3	231.8	Nano-Fm	600	263.5	56.1	168.3
Nano-Tn	750	221.0	70.5	264.5	Nano-Fn	750	402.6	46.3	173.7

The rationale for the sample labels appearing in the table: "CP" indicates iowaite synthesized by conventional co-precipitation method and "Nano" nanocrystalline iowaite generated via fast coprecipitation followed by hydrothermal treatment; the capital letters "T" and "F" refer to arsenite and arsenate, respectively; the lowercase letters following "T" or "F" (a, b, c, d, e, f, g, h, i, j, k, l, m and n) represent the initial arsenic concentrations of the solutions (a: 150 μg/L; b: 300 μg/L; c: 750 μg/L; d: 1500 μg/L; e: 3000 μg/L; f: 7500 μg/L; g: 15 mg/L; h: 30 mg/L; i: 75 mg/L; j: 150 mg/L; k: 300 mg/L; l: 450 mg/L; m: 600 mg/L; n: 750 mg/L).

Table S5. Percentages of remained Nano-iowaite in the solid samples after reaction with 0.2 mmol/L arsenic (equimolarly mixed arsenate and arsenite) solutions at initial pHs of 2, 3, 4, 5, and 6.

**Initial solution pH**	**Solution arsenic concentration at equilibrium (mg/L)**	**Percentages of remained Nano-iowaite after reaction (%)**
2.0	0.035	96.99
3.0	0.113	99.42
4.0	0.121	99.62
5.0	0.119	99.63
6.0	0.106	99.59

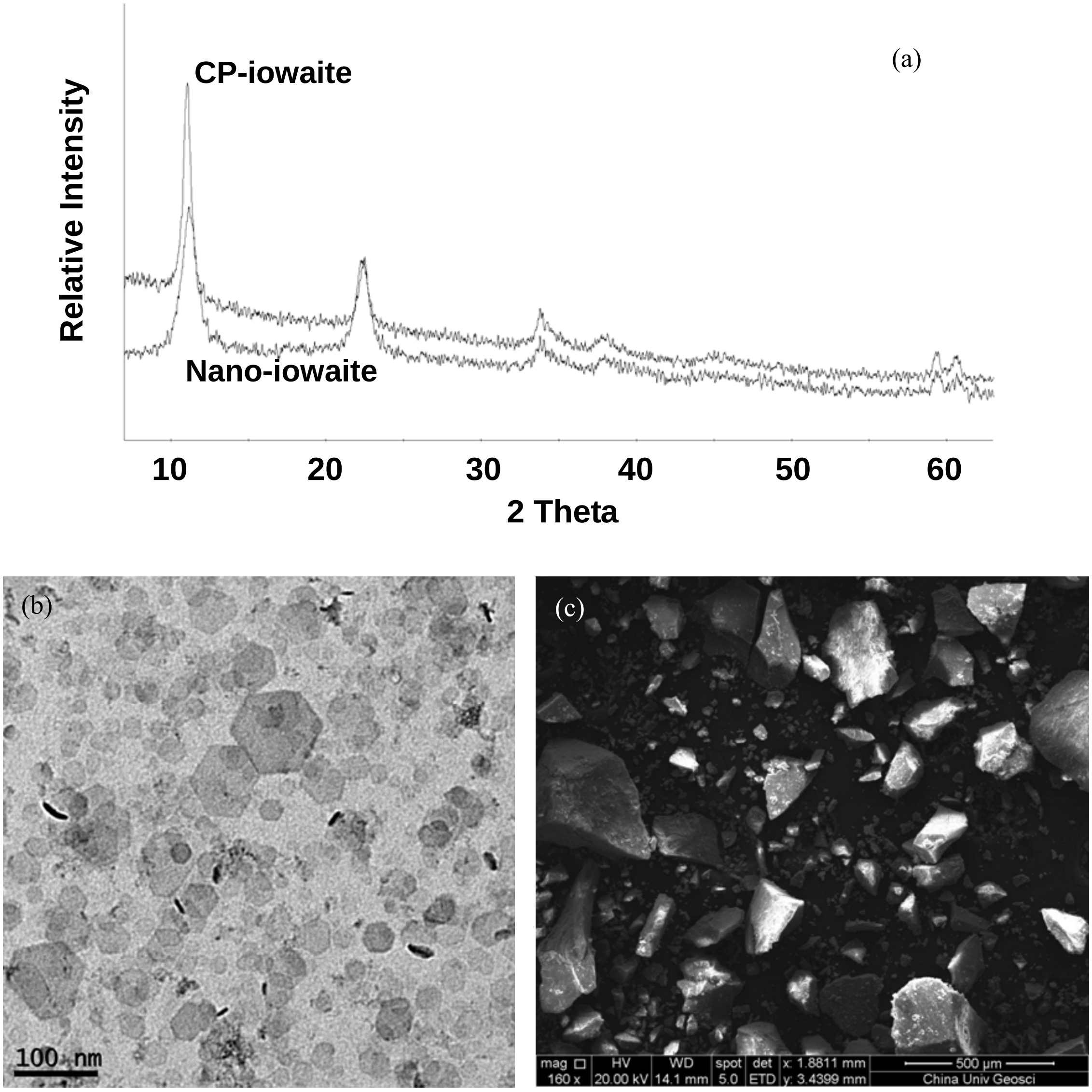

Figure S1. (a) XRD patterns of synthetic CP-iowaite and Nano-iowaite obtained using Cu Kα radiation, (b) TEM image of synthetic Nano-iowaite, and (c) SEM image of synthetic CP-iowaite.

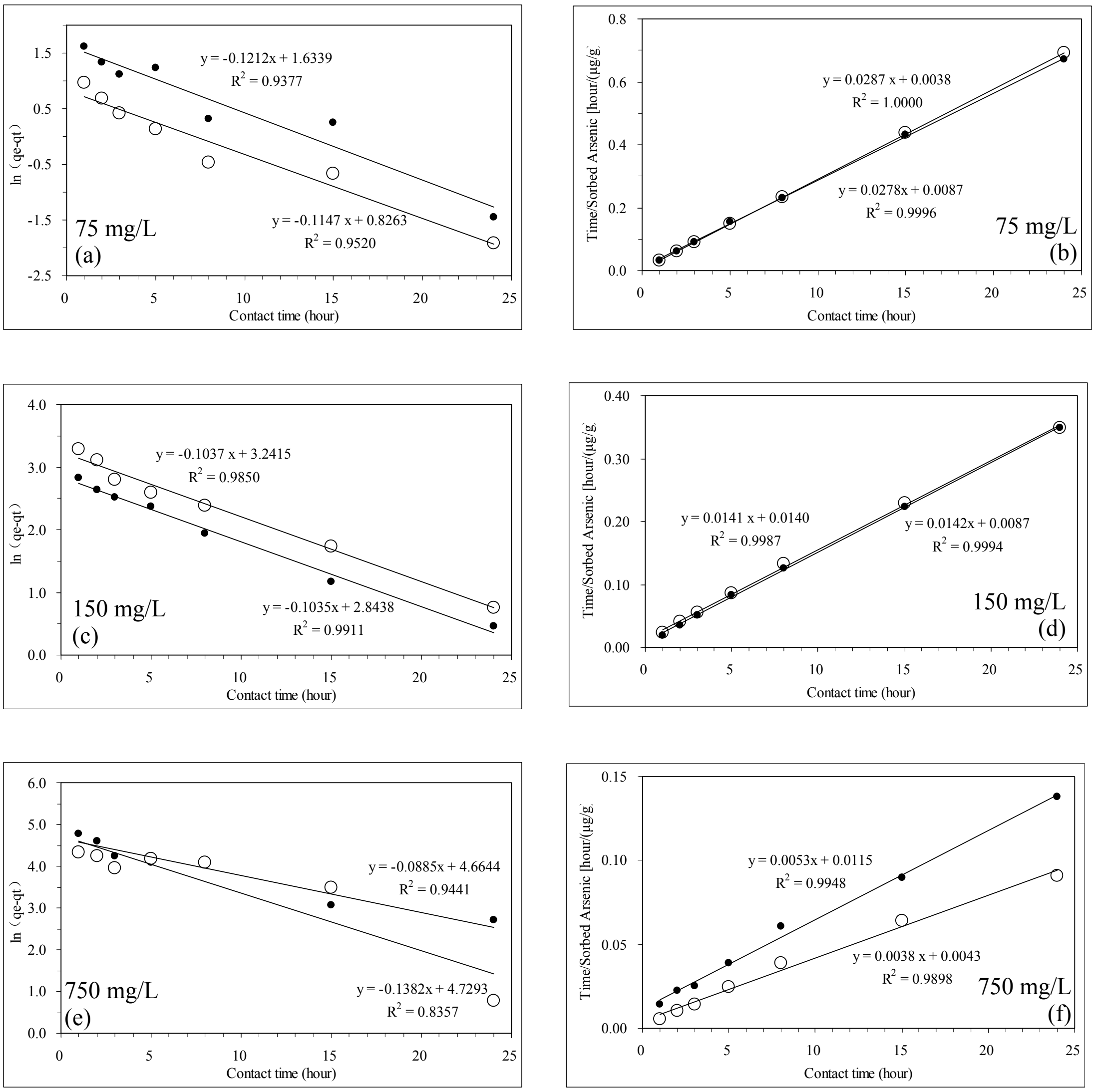

Figure S2. Fit of kinetics data for arsenic removal by Nano-iowaite to the Lagergren first-order model (a, c and e) and the pseudo second-order model (b, d and f). Legends: ○, arsenite; ●, arsenate.

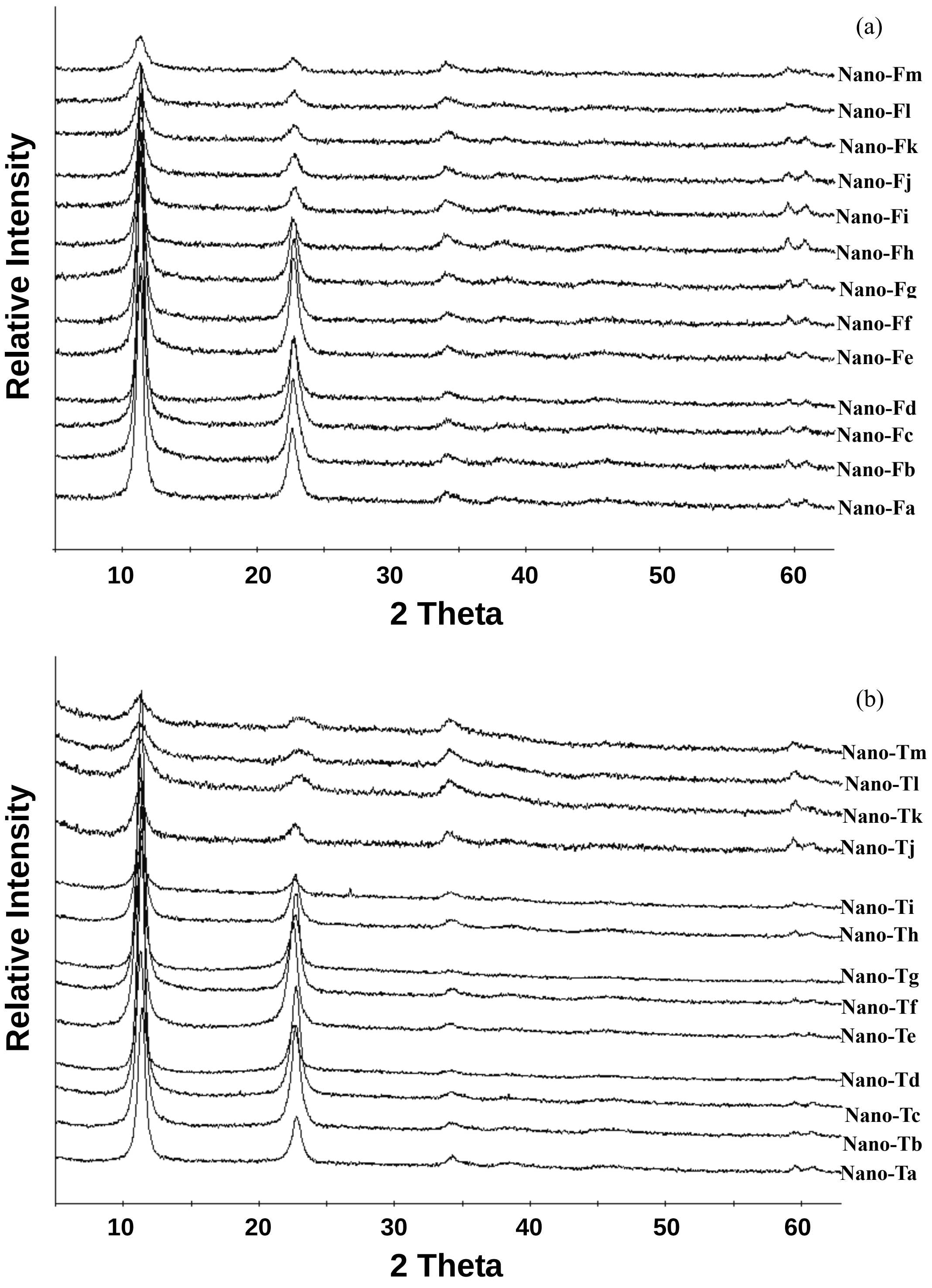

Figure S3. XRD patterns of Nano-iowaite after reacting with arsenate (a) and arsenite (b) solutions with initial concentrations lower than 750 mg/L. The sample numbers beside the curves indicate the initial concentrations of arsenate or arsenite in solution. Specifically, the lowercase letters following "F" or "T", i.e. a, b, c, d, e, f, g, h, i, j, k, l, and m, represent initial arsenic concentrations of 150 μg/L, 300 μg/L, 750 μg/L, 1500 μg/L, 3000 μg/L, 7500 μg/L, 15 mg/L, 30 mg/L, 75 mg/L, 150 mg/L, 300 mg/L, 450 mg/L, and 600 mg/L, respectively.

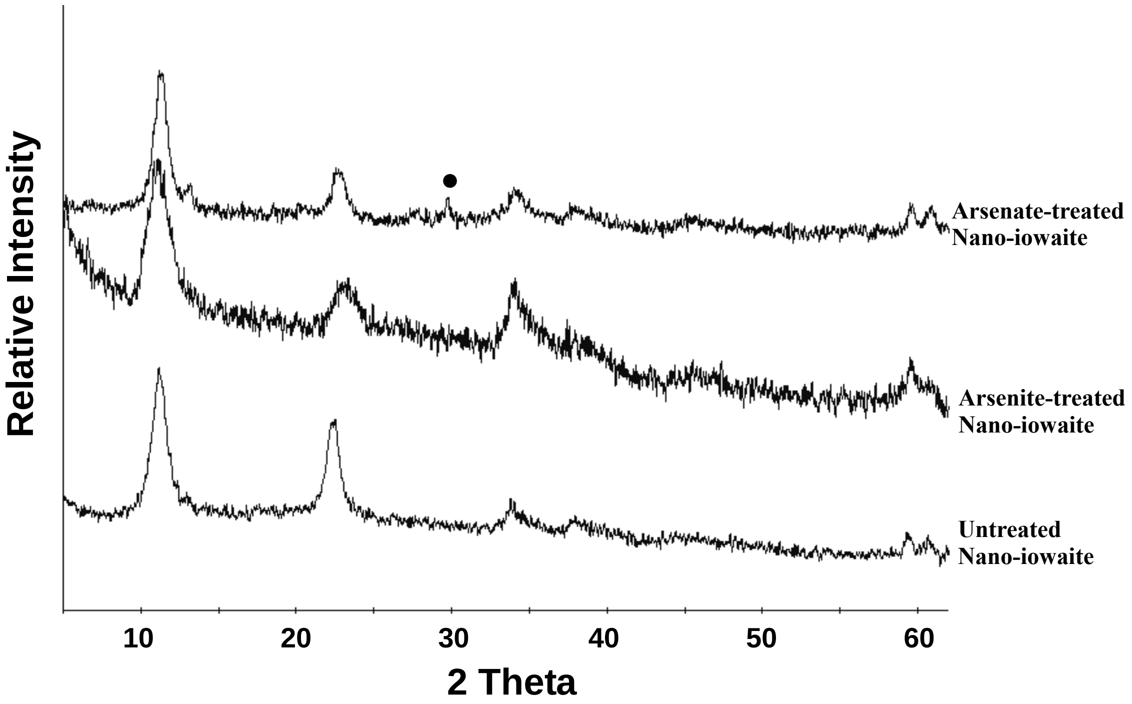

Figure S4. XRD patterns of the Nano-iowaite samples before and after reaction with arsenate and arsenite solutions with an initial concentration of 750 mg/L. ●: angelellite ($Fe_4(AsO_4)_2O_3$) or hornesite ($Mg_3(AsO_4)_2 \cdot 8H_2O$).

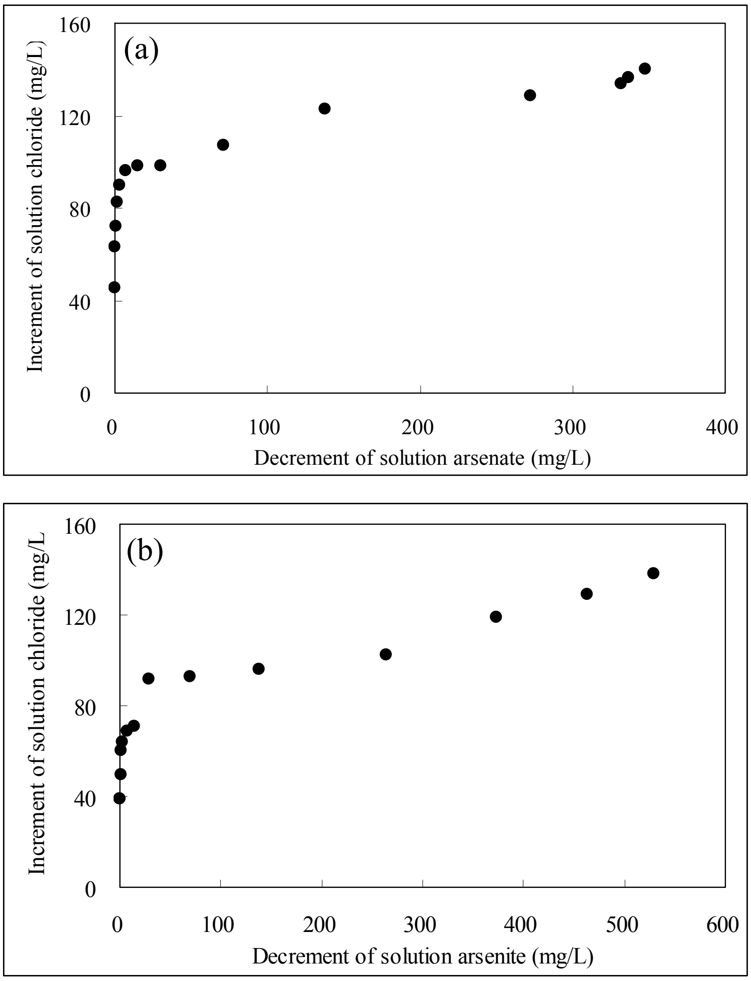

Figure S5. Variations in the increment of chloride vs. the decrement of arsenate (a) or arsenite (b) in the solutions reacted with Nano-iowaite.

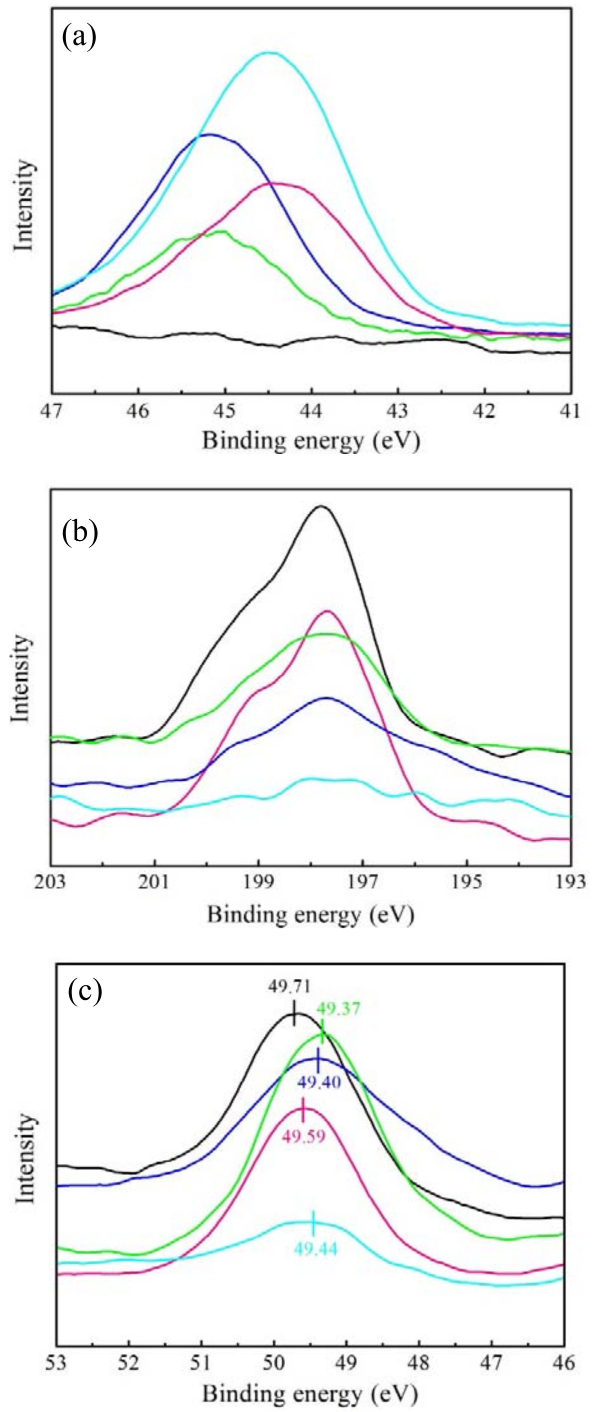

Figure S6. XPS spectra of As 3d (a), Cl 2p (b) and Mg 2p (c) for unreacted Nano-iowaite samples and arsenic-loaded samples that have been reacted with 150 and 750 mg/L arsenic solutions. —— unreacted Nano-iowaite sample; —— Nano-iowaite sample reacted with 150 mg/L arsenate solution (Nano150F); —— Nano-iowaite sample reacted with 750 mg/L arsenate solution (Nano750F); —— Nano-iowaite sample reacted with 150 mg/L arsenite solution (Nano150T); —— Nano-iowaite sample reacted with 750 mg/L arsenite solution (Nano750T).

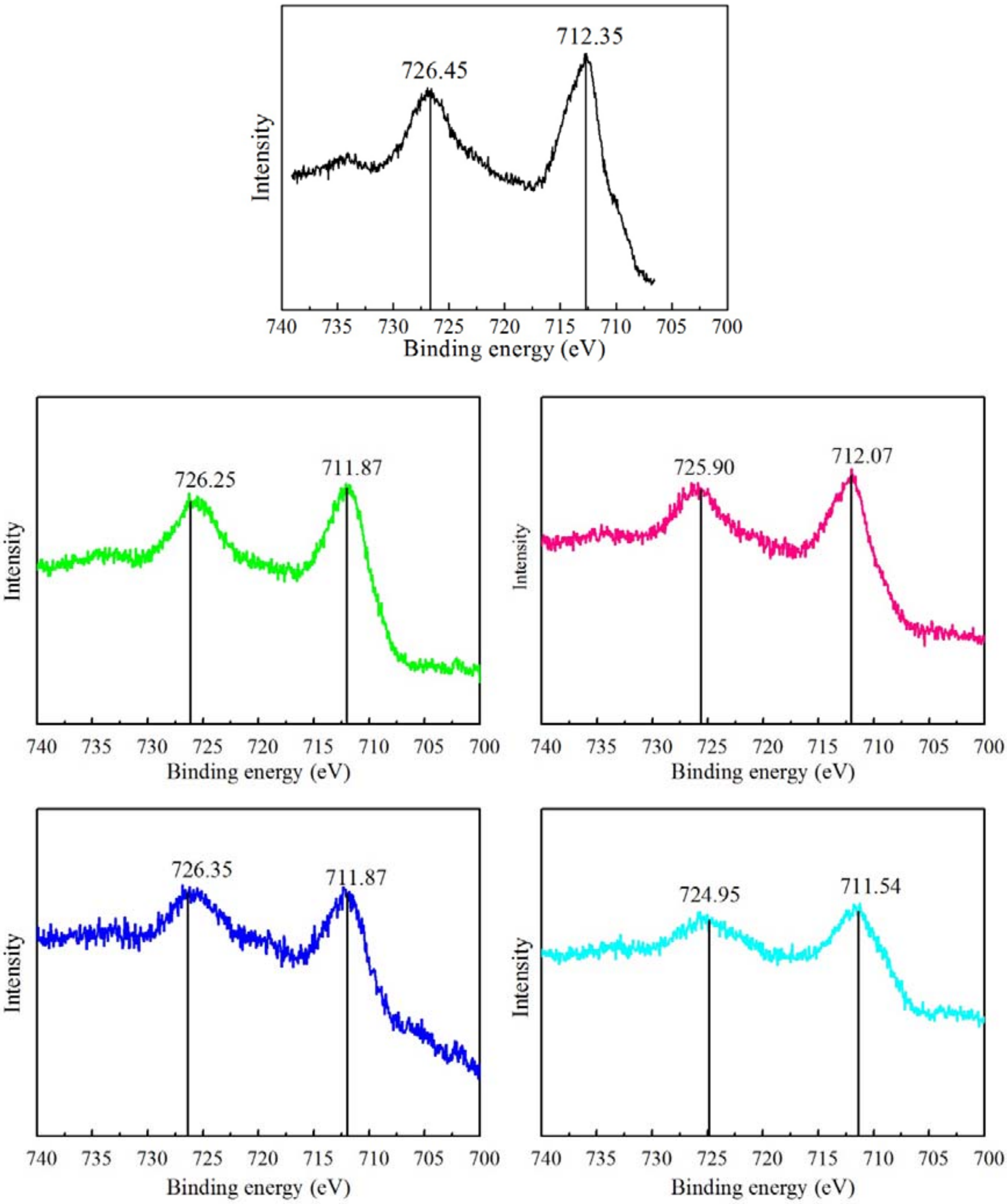

Figure S7. XPS spectra of Fe 2p for unreacted Nano-iowaite samples and arsenic-loaded samples that have been reacted with 150 and 750 mg/L arsenic solutions. —— unreacted Nano-iowaite sample; —— Nano-iowaite sample reacted with 150 mg/L arsenate solution (Nano150F); —— Nano-iowaite sample reacted with 750 mg/L arsenate solution (Nano750F); —— Nano-iowaite sample reacted with 150 mg/L arsenite solution (Nano150T); —— Nano-iowaite sample reacted with 750 mg/L arsenite solution (Nano750T).

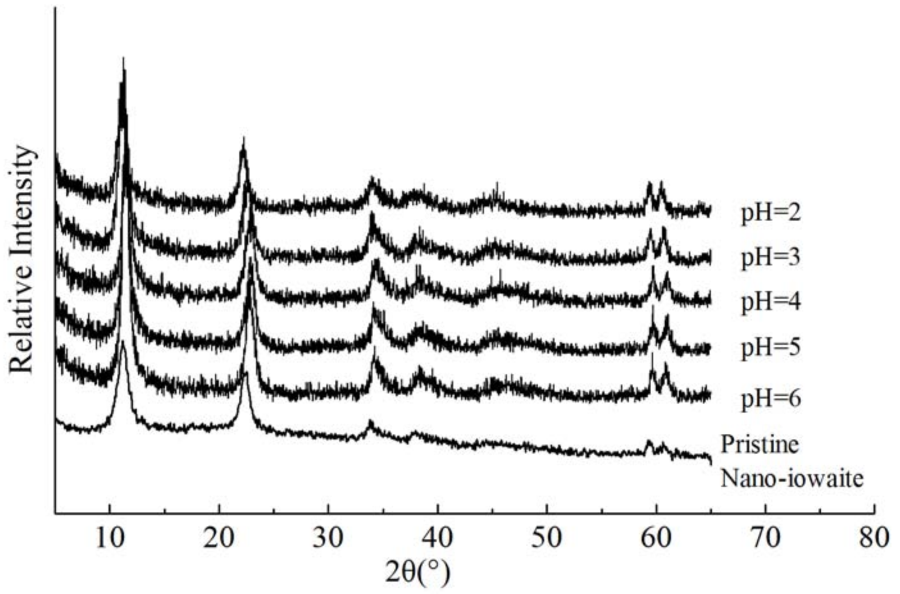

Figure S8. XRD patterns of the Nano-iowaite samples after reaction with 0.2 mmol/L arsenic (equimolarly mixed arsenate and arsenite) solutions at initial pHs of 2, 3, 4, 5, and 6.

References

1. Lagergren, S. About the theory of so-called adsorption of soluble substances. *Kungliga Svenska Vetenskapsakademiens Handlingar* **24**, 1-39 (1898).
2. Ho, Y. S. & McKay, G. Pseudo-second order model for sorption processes. *Process Biochem* **34**, 451-465 (1999).
3. Cao, Y. *et al.* Application of calcined iowaite in arsenic removal from aqueous solution. *Applied Clay Science* **126**, 313-321 (2016).
4. Goh, K.-H., Lim, T.-T. & Dong, Z. Enhanced arsenic removal by hydrothermally treated nanocrystalline Mg/Al layered double hydroxide with nitrate intercalation. *Environmental science & technology* **43**, 2537-2543 (2009).
5. Vaishya, R. C. & Gupta, S. K. Modeling Arsenic (V) Removal from Water by Sulfate Modified Iron - Oxide Coated Sand (SMIOCS). *Separation Science and Technology* **39**, 645-666 (2005).
6. Ona-Nguema, G., Morin, G., Juillot, F., Calas, G. & Brown, G. E. EXAFS analysis of arsenite adsorption onto two-line ferrihydrite, hematite, goethite, and lepidocrocite. *Environmental science & technology* **39**, 9147-9155 (2005).
7. Daus, B., Wennrich, R. & Weiss, H. Sorption materials for arsenic removal from water:: a comparative study. *Water Research* **38**, 2948-2954 (2004).
8. Singh, D., Prasad, G., Rupainwar, D. & Singh, V. As (III) removal from aqueous solution by adsorption. *Water, Air, and Soil Pollution* **42**, 373-386 (1988).
